# A novel immunogenic cell death-related classification indicates the immune landscape and predicts clinical outcome and treatment response in acute myeloid leukemia

**DOI:** 10.1186/s12935-024-03326-0

**Published:** 2024-04-16

**Authors:** Fangmin Zhong, Shuyang He, Ni Guo, Luyi Shi, Linlin Zhang, Hua Jin, Guangyao Kong

**Affiliations:** 1 Department of Hematology, The Second Affiliated Hospital of Xi’an Jiaotong University, Xi’an, Shaanxi People’s Republic of China; 2 Jiangxi Province Key Laboratory of Laboratory Medicine, Jiangxi Provincial Clinical Research Center for Laboratory Medicine, Department of Clinical Laboratory, The Second Affiliated Hospital, Jiangxi Medical College, Nanchang University, Nanchang, Jiangxi China; 3 Queen Mary School of Nanchang University, Nanchang, Jiangxi People’s Republic of China; 4 Precision Medical Institute, The Second Affiliated Hospital of Xi’an Jiaotong University, Xi’an, Shaanxi People’s Republic of China; 5 National and Local Joint Engineering Research Center of Biodiagnosis and Biotherapy, The Second Affiliated Hospital of Xi’an Jiaotong University, Xi’an, Shaanxi People’s Republic of China; 6 Key Laboratory of Surgical Critical Care and Life Support, Xi’an Jiaotong University, Xi’an, Shaanxi People’s Republic of China

## Abstract

**Background:**

Immunogenic cell death (ICD) is closely related to anti-tumor therapy and regulates the tumor microenvironment (TME). This study aims to explore the molecular characteristics of ICD in acute myeloid leukemia (AML) and to analyze the value of ICD-related biomarkers in TME indication, prognosis prediction, and treatment response evaluation in AML.

**Methods:**

Single-sample gene set enrichment analysis was used to calculate the ICD score. LASSO regression was used to construct a prognostic risk score model. We also analyzed differences in clinical characteristics, immune landscape, immunotherapy response, and chemotherapy sensitivity between high-risk and low-risk patients.

**Results:**

This study identified two ICD-related subtypes and found significant heterogeneity in clinical prognosis, TME, and immune landscape between different ICD subtypes. Subsequently, a novel ICD-related prognostic risk score model was developed, which accurately predicted the prognosis of AML patients and was validated in nine AML cohorts. Moreover, there were significant correlations between risk scores and clinicopathological factors, somatic mutations, TME characteristics, immune cell infiltration, immunotherapy response, and chemosensitivity. We further validated the model gene expression in a clinically real-world cohort.

**Conclusions:**

The novel ICD-related signatures identified and validated by us can serve as promising biomarkers for predicting clinical outcomes, chemotherapy sensitivity, and immunotherapy response in AML patients, guiding the establishment of personalized and accurate treatment strategies for AML.

**Graphical Abstract:**

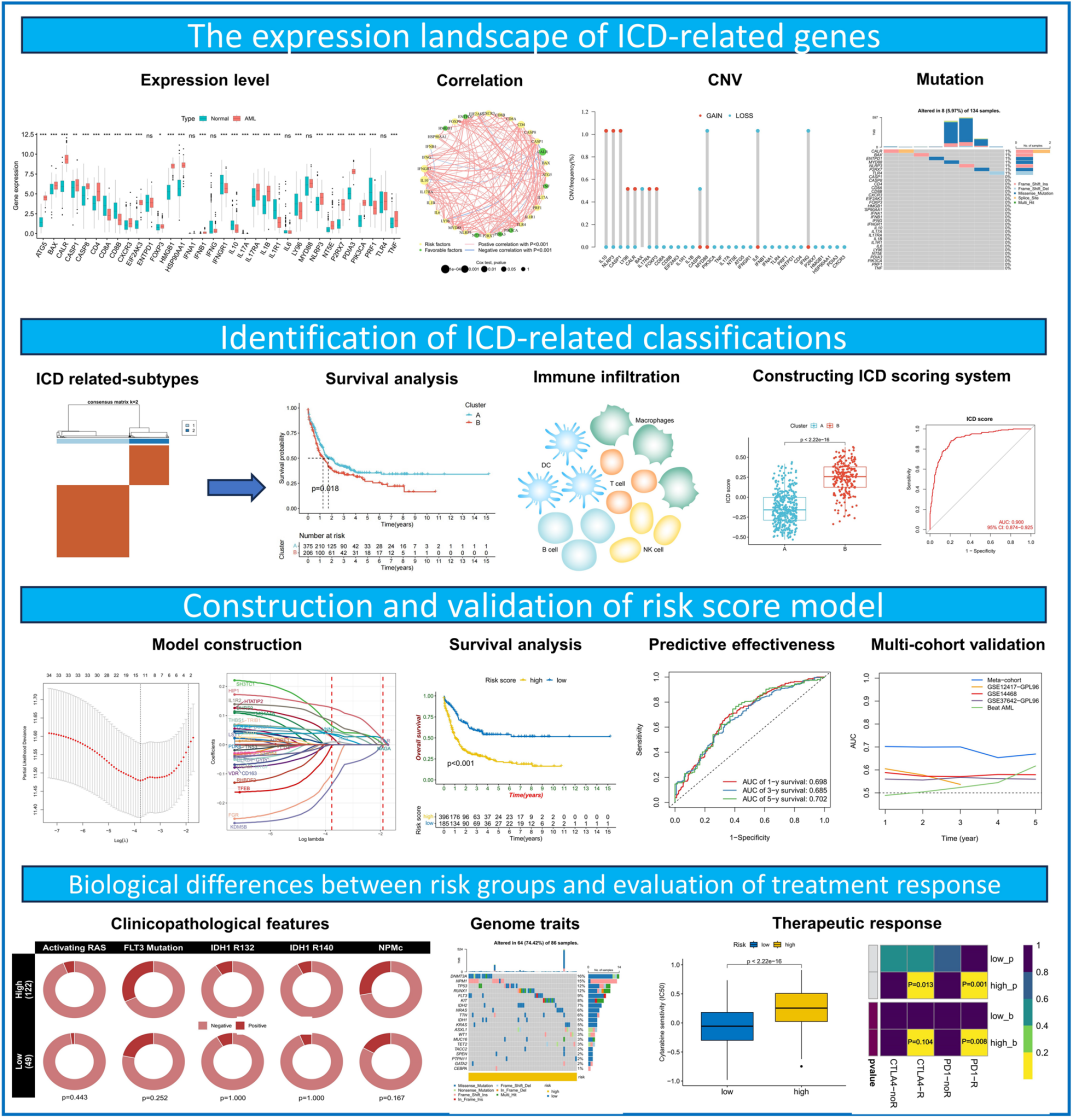

**Supplementary Information:**

The online version contains supplementary material available at 10.1186/s12935-024-03326-0.

## Introduction

Acute myeloid leukemia (AML) is a hematological tumor with malignant proliferation of hematopoietic stem cells [1]. AML is heterogeneous due to complex acquired somatic mutations and genomic mutations [2]. Although new targeted drugs have made great achievements in the individualized and precise treatment of AML, the long-term survival and complete remission (CR) rates of patients are still unsatisfactory [3]. Therefore, there is an urgent need to find new reliable biomarkers for AML diagnosis, prognostic stratification, and personalized targeted therapies.

Immunogenic cell death (ICD) is a unique form of tumor cell death characterized by the transformation of tumor cells from a nonimmunogenic state to immunogenic, thereby activating anti-tumor immune effects in vivo, leading to tumor cell death [4]. The main process of ICD is that dying tumor cells release damage-associated molecular patterns (DAMPs), activate and recruit antigen-presenting cells, and further activate T cells to produce adaptive immune responses against tumor antigens [5]. An increasing number of studies have confirmed that ICD induction is a particularly effective treatment for tumors that are resistant to conventional therapies [6, 7]. For example, the combination of dendritic cells and doxorubicin induces immunogenic cell death and plays an antitumor role in osteosarcoma [8]. As a hematological tumor, the tumor microenvironment (TME) of AML contains a large number of immune cells. Induced AML cells ICD can more directly promote the immune cells play antitumor immune effect, showing a potential treatment feasibility. It has been shown that increased exposure of DAMPs such as calreticulin on the plasma membrane of AML malignant blast cells is a novel powerful prognostic biomarker in AML patients, reflecting activation of clinically relevant AML-specific immune responses [9]. Moreover, chemotherapy-treated dying AML cells also release ATP to cause immunosuppression by increasing the number of regulatory T cells and tolerogenic dendritic cells [10]. All the evidence reflects that AML is closely related to ICD. However, the relevance of ICD-related genes (ICDRGs) to clinical prognosis and anticancer mechanisms in AML remains unclear. Therefore, a comprehensive understanding of the molecular features of ICDRGs can provide insight into the causes of AML heterogeneity.

In this project, we analyzed the expression profiles of ICDRGs and constructed ICD-related molecular subtypes to explore the underlying mechanisms of ICD. Subsequently, we developed a novel risk score model based on the molecular features of ICD subtypes to help predict clinical outcomes, immune landscape, immunotherapy response, and chemotherapy sensitivity in AML patients. More importantly, we validated the analytical results by in vitro experiments. These results contribute to the prognosis prediction of AML and provide more individualized and effective treatment strategies for AML patients.

## Methods

### Data acquisition and preprocessing

A total of nine AML cohorts with 2059 AML bone marrow samples containing clinical survival information were included in this study. There were eight Gene Expression Omnibus (GEO) cohorts (TCGA-LAML (GSE68833), GSE10358, GSE12417-GPL96, GSE12417-GPL570, GSE37642-GPL570, GSE37642-GPL96, GSE71014, GSE14688) and the Beat AML cohort. For GEO cohorts on the Affymetrix platform, we downloaded the raw "CEL" files and normalized them using a robust multiarray averaging (RMA) method. For GEO cohorts on other platforms, we directly download the already standardized matrix files. RNA-sequencing data of the Beat AML cohort were transformed into transcripts per million (TPM) values. Considering the more complete number of genes tested in GSE68833, GSE10358, GSE12417-GPL570, GSE37642-GPL570, and GSE71014 cohorts, we used the "combat" algorithm of the "sva" package for batch correction. A meta-cohort was formed for subsequent analysis. Data of somatic mutation, and gene copy number were downloaded from the TCGA database (https://portal.gdc.cancer.gov/). The definition of overall survival (OS) was the duration from diagnosis to death resulting from any cause or until the censoring date of the last follow-up. Patients were excluded if they experienced premature death (OS < 15 days) or had missing follow-up data. Additional file 1: Table S1 shows sample information for all cohorts.

### Identification of ICD-related molecular subtypes by consensus clustering

The list of ICDRGs was obtained from previous studies [11]. We used the "ConsensusclusterPlus" software package to conduct consensus clustering based on the expression of these genes to identify ICD-related molecular subtypes. To obtain stable and reliable classification results, we iterated 1000 times. t-distributed stochastic neighbor embedding (t-SNE) was used to validate the classification [12]. In addition, we performed gene set variation analysis (GSVA) and gene set enrichment analysis (GSEA) were used to analyze the differences in biological processes between the different ICD-related molecular subtypes [13].

### Immune cell infiltration and TME assessment

The CIBERSORT algorithm was used to calculate the proportion of 22 immune cells in AML samples [14]. The ESTIMATE algorithm was used to evaluate the immune, stromal, and ESTIMATE scores of each AML sample [15].

### Identification of differentially expressed genes (DEGs) between molecular subtypes

The "limma" package [16] was used to identify DEGs between molecular subtypes with filtering thresholds as follows: (1) FDR < 0.05; (2) |log fold change (FC)|> 1. Then, the functions of DEGs were analyzed by performing Kyoto Encyclopedia of Genes and Genomes (KEGG) pathway analysis and Gene Ontology (GO) annotation using the "clusterProfiler" package.

### Construction of ICD score system and risk score model

To quantify ICD-related molecular subtypes, we used single-sample gene set enrichment analysis (ssGSEA) to calculate ICD scores for AML samples according to the expression of ICDRGs. For the construction of the risk score model, genes significantly associated with the prognosis of AML patients were identified from the DEGs of molecular subtypes (P < 0.001). Least absolute shrinkage and selection operator (LASSO)-stepwise multivariate Cox regression analysis was used to construct the risk score model.

$$Risk\, score= \sum\limits_{1}^{i}(Coefi*ExpGenei),$$

where i is the model gene, "Coef" and "ExpGene" are the non-0 regression coefficient and the expression value of it. Based on the optimal cut-off value, the AML cohort was divided into high- and low-risk groups for subsequent analysis.

### Prediction of immunotherapy response and chemotherapy sensitivity

The "pRRophetic" package was used to predict the half maximal inhibitory concentration (IC50) of AML samples to commonly used therapeutic agents [17]. A smaller IC50 value indicates a higher therapeutic sensitivity of the drug. We used the SubMap algorithm (https://cloud.genepattern.org/gp) to predict the response of different risk groups to immune checkpoint inhibitor therapy with anti-PD-1 and anti-CTLA4. Moreover, to evaluate the immune escape level of tumor cells, we used the Tumor Immune Dysfunction and Exclusion (TIDE) website (http://tide.dfci.harvard.edu/) to calculate the TIDE score of AML samples.

### Collection of clinical samples from patients with myeloid leukemia

This study was approved by the Ethics Committee of the Second Affiliated Hospital of Nanchang University, and all procedures were in accordance with regulations. Five samples from patients with newly diagnosed chronic myeloid leukemia without any previous treatment, five samples from patients in blast crisis, and five normal samples from healthy volunteers were collected according to the World Health Organization classification of tumors of hematopoietic and lymphoid tissues. Details of sample collection, next-generation sequencing, and processing procedures are provided in our previous report [18].

### Statistical analysis

Statistical analyses were performed with the use of R software, version 4.1.2. The Wilcoxon test was used to compare the differences between the two groups, and the Kruskal–Wallis test was used to analyze the differences among the multiple groups. P < 0.05 was considered statistically significant (*P < 0.05, **P < 0.01, ***P < 0.001).

## Results

### The expression landscape of ICDRGs

**Fig. 1:**
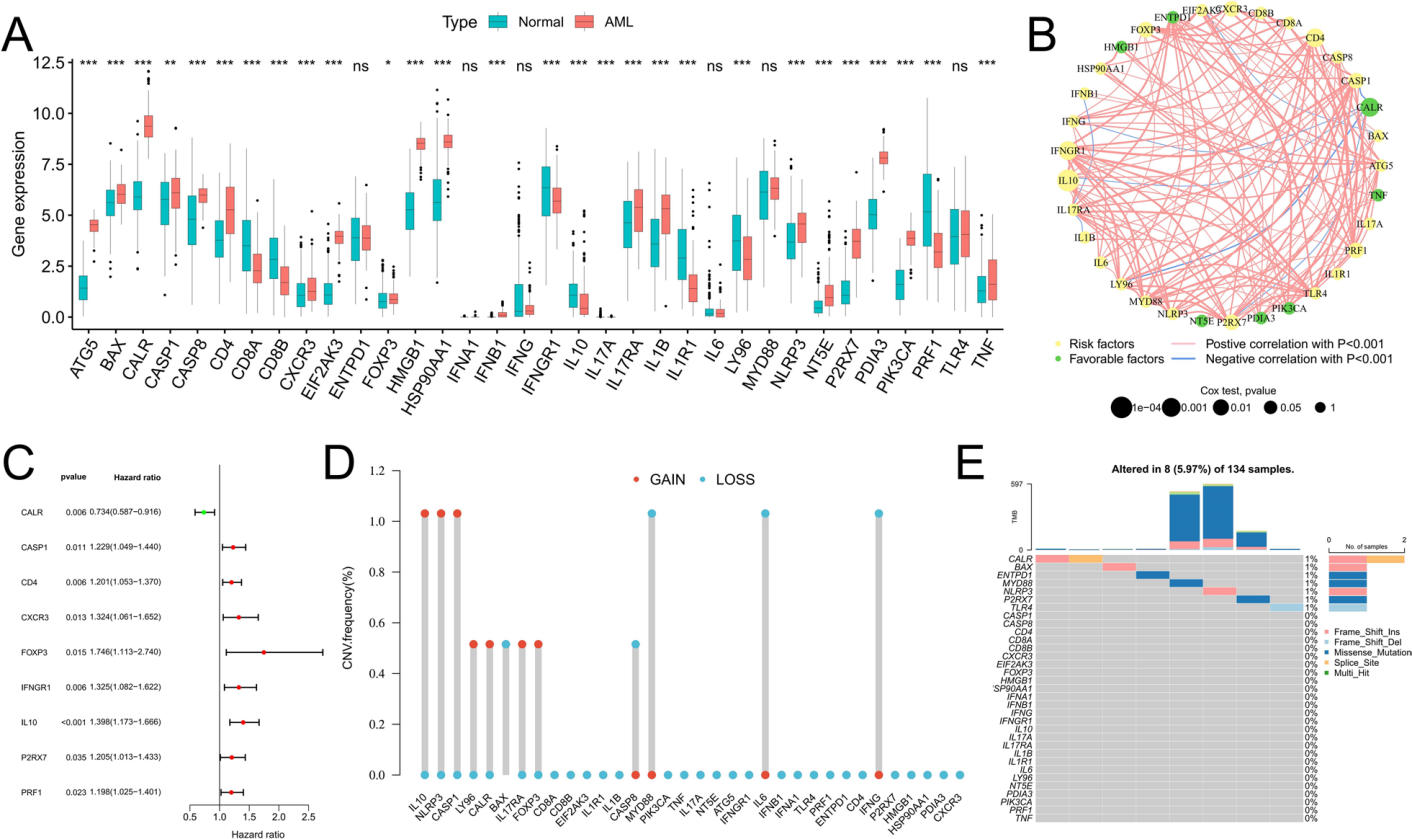
Genetic characteristics of ICDRGs. **A** The interaction of ICDRGs in AML patients and its relationship with prognosis. **B** Differential expression analysis of ICDRGs between AML samples and normal samples. **C** Univariate Cox regression analysis was used to identify ICDRGs significantly associated with prognosis. **D** CNV frequency of ICDRGs in the TCGA-LAML cohort. **E** Somatic mutations of ICDRGs in the TCGA-LAML cohorts

The results of the differential analysis showed that most ICDRGs such as ATG5, BAX, and CALR were up-regulated in AML samples (Fig. 1A), and some ICDRGs such as CD8A, CD8B, and IFNGR1 were down-regulated. There was no significant difference in the expression of ICDRGs such as ENTPD1, IFNA1, and IFNG between AML and normal samples. Univariate Cox regression analysis showed that most of the ICDRGs were risk factors for AML and were associated with poor prognosis of patients (Fig. 1B). Moreover, CALR, CASP1, CD4, CXCR3, FOXP3, IFNGR1, IL10, P2RX7, and PRF1 were significantly correlated with the prognosis of AML patients (p < 0.05) (Fig. 1C). We also found that almost all ICDRGs were positively correlated with each other except CALR, which was negatively correlated with multiple ICDRGs (Fig. 1B). Copy number variation analysis showed that the frequency of copy number gain of IL10, NLRP3, and CASP1 was significantly increased, while the frequency of copy number loss of MYD88, IL6, and IFNG was significantly increased (Fig. 1D). Somatic mutation analysis showed that the overall mutation rate of ICDRGs was low (Fig. 1E).

### Identification of ICD-related classifications in AML

**Fig. 2:**
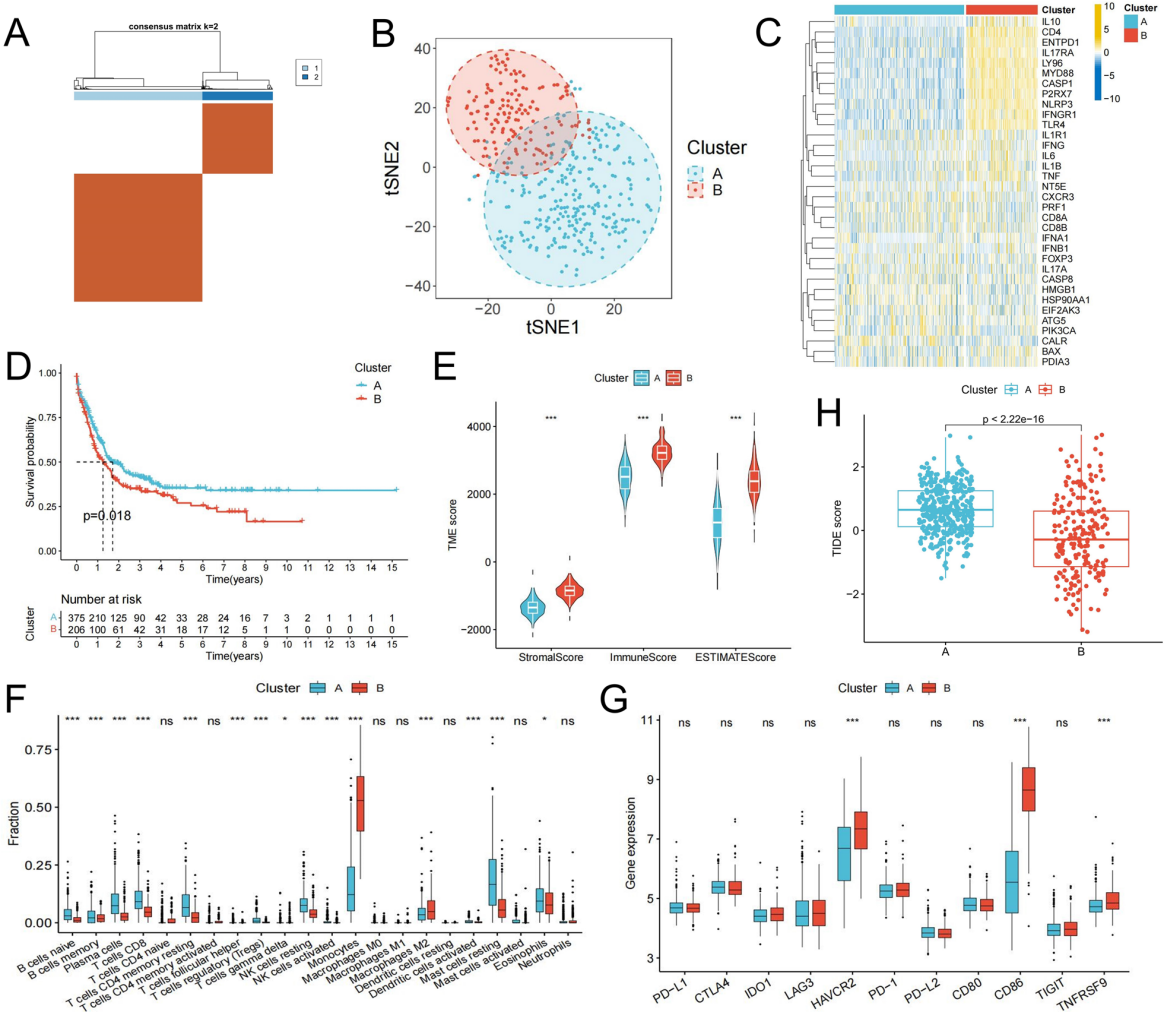
Identification of ICD-related molecular subtypes and analysis of differences in TME characteristics between subtypes. **A** Two molecular subtypes were identified by consensus clustering. **B** The TSNE algorithm was used to verify the accuracy of molecular subtypes. **C** Heatmap shows the expression characteristics of ICDRGs between subtypes. **D** Survival analysis between subtypes. **E**–**H** Differences in TME scores (**E**), immune cell infiltration (**F**), immune checkpoint expression (**G**), and TIDE score (**H**) between subtypes

To further explore the expression characteristics of ICDRGs in AML, consensus clustering algorithm was used to classify the meta-cohort. The results showed that when the number of clusters was 2, the classification effect was the best. 581 patients with AML were divided into two kinds of ICD-related molecular subtypes: Cluster A (n = 375) and Cluster B (n = 206) (Fig. 2A). The t-SNE algorithm showed significant differences between these two ICD-related molecular subtypes, which confirmed the reliability of the classification (Fig. 2B). The heatmap showed that most ICDRGs were upregulated in Cluster B compared with Cluster A (Fig. 2C). Kaplan–Meier curve analysis also showed that there was a significant difference in overall survival between the two subtypes, and the prognosis of patients in Cluster A was better (Fig. 2D). Thus, the two ICD-related molecular subtypes identified based on ICDRG expression are significantly different.

### Analysis of TME differences between classifications

We further explored TME differences between the two ICD-related subtypes. TME analysis showed that Cluster B had higher TME scores, including stromal, immune, and ESTIMATE scores (Fig. 2E). The results of immune infiltration analysis showed that adaptive immune cells such as naive and memory B cells, plasma cells, CD8+ T cells, resting CD4+ T cells, regulatory T cells (Tregs), and innate immune cells such as resting NK cells and mast cells in Cluster A exhibited higher infiltration levels than those in Cluster B. Monocytes and M2 macrophages were mainly enriched in Cluster B (Fig. 2F). Therefore, we hypothesized that the better prognosis of Cluster A patients might be related to their higher proportion of anti-tumor immune cell infiltration. The expression levels of key immune checkpoints such as PD-1, PD-L1, and CTLA4 were not significantly different between the two groups, while HAVCR2, CD86, and TNFRSF9 were up-regulated in Cluster B (Fig. 2G). We further calculated the TIDE score to evaluate the immune escape ability of tumor cells. Strikingly, the TIDE score of Cluster A was significantly higher than that of Cluster B (Fig. 2H), suggesting that there may be some suppression of anti-tumor immune effects in Cluster A.

### Constructing an ICD scoring system to explore the potential correlation between ICD and TME

**Fig. 3:**
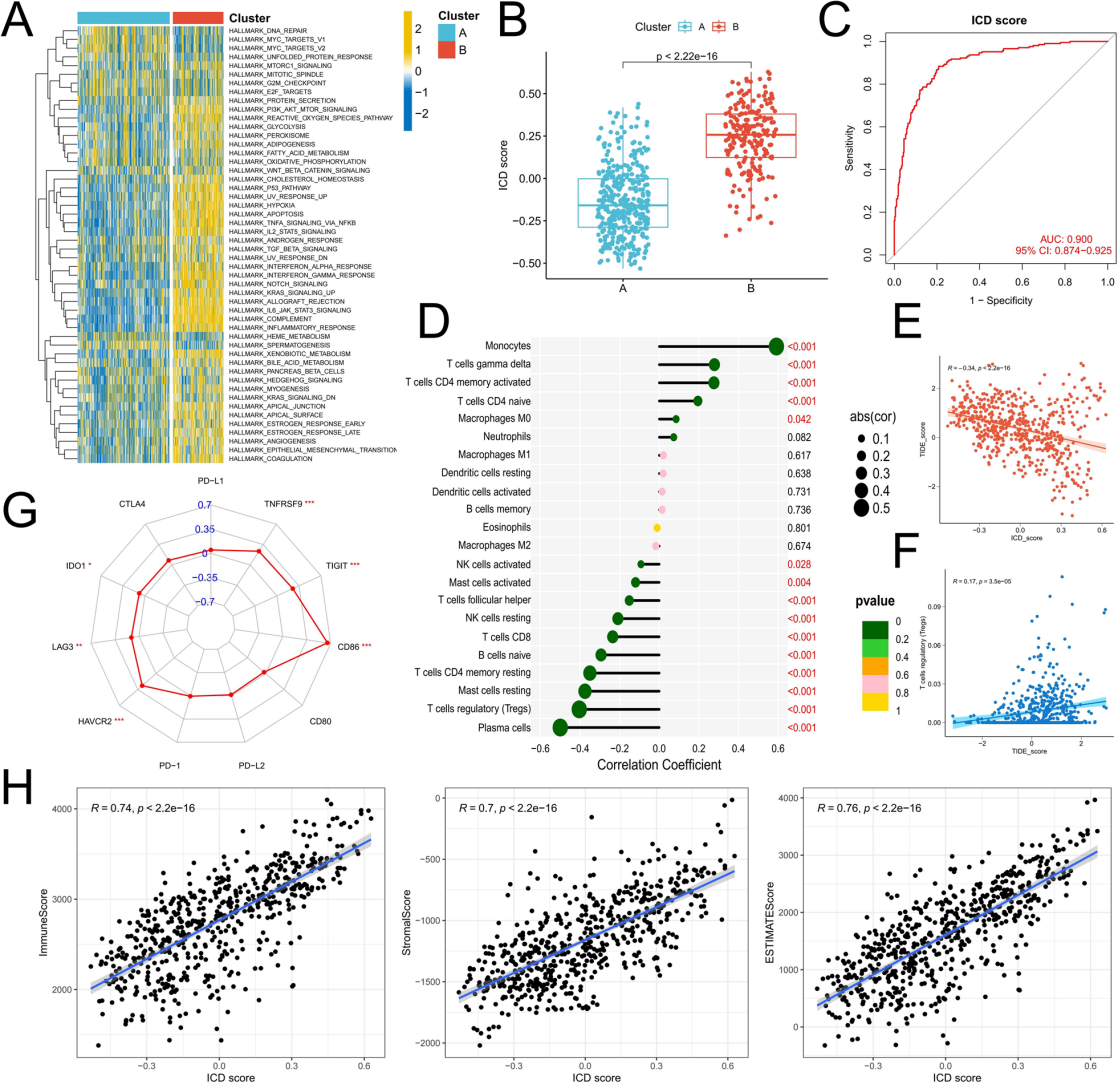
Construction of ICD scoring system and correlation analysis with TME. **A** Differences in enrichment scores of tumor marker gene sets between ICD molecular subtypes. **B** Differences in ICD score between subtypes. **C** ROC curve analysis was used to verify the ability of the ICD score to discriminate subtypes. **D** Correlation between ICD score and immune cell infiltration. **E**, **F** Correlation of TIED score with Treg infiltration and ICD score. **G** Correlations of the immune checkpoint genes with ICD score. **H** Correlations of the ICD score with immune score, stromal score, and ESTIMATE score

Subsequently, we compared the differences in enrichment scores between tumor marker pathways between the subtypes, and the activity of these pathways was essentially higher in Cluster B, indicating that the subtype had more active signaling (Fig. 3A). In addition, we used the ssGSEA algorithm to determine the ICD score to evaluate ICD activity and explain the interaction between ICD and TME. First of all, the ICD score quantified ICD subtypes well, and the ICD score of Cluster B was significantly higher than that of Cluster A (Fig. 3B). The ROC curve also confirmed that the ICD score could effectively distinguish ICD subtypes (AUC = 0.900) (Fig. 3C). Second, high ICD scores were associated with lower infiltration of anti-tumor immune cells (Fig. 3D). Interestingly, the infiltrating proportion of Tregs was significantly negatively correlated with the ICD score and positively correlated with the TIDE score (Fig. 3E, F). Therefore, we hypothesized that infiltration of Tregs might be involved in the immunosuppression of Cluster A. Finally, the ICD score was also significantly positively correlated with the expression of immune checkpoints such as HAVCR2, CD86, TNFRSF9, TIGIT, IDO1, and LAG3 (Fig. 3G). TIDE also had a strong correlation with TME scores (Fig. 3H). These findings indicated that ICD could participate in regulating the TME in AML.

### Analysis of biological differences between classifications

**Fig. 4:**
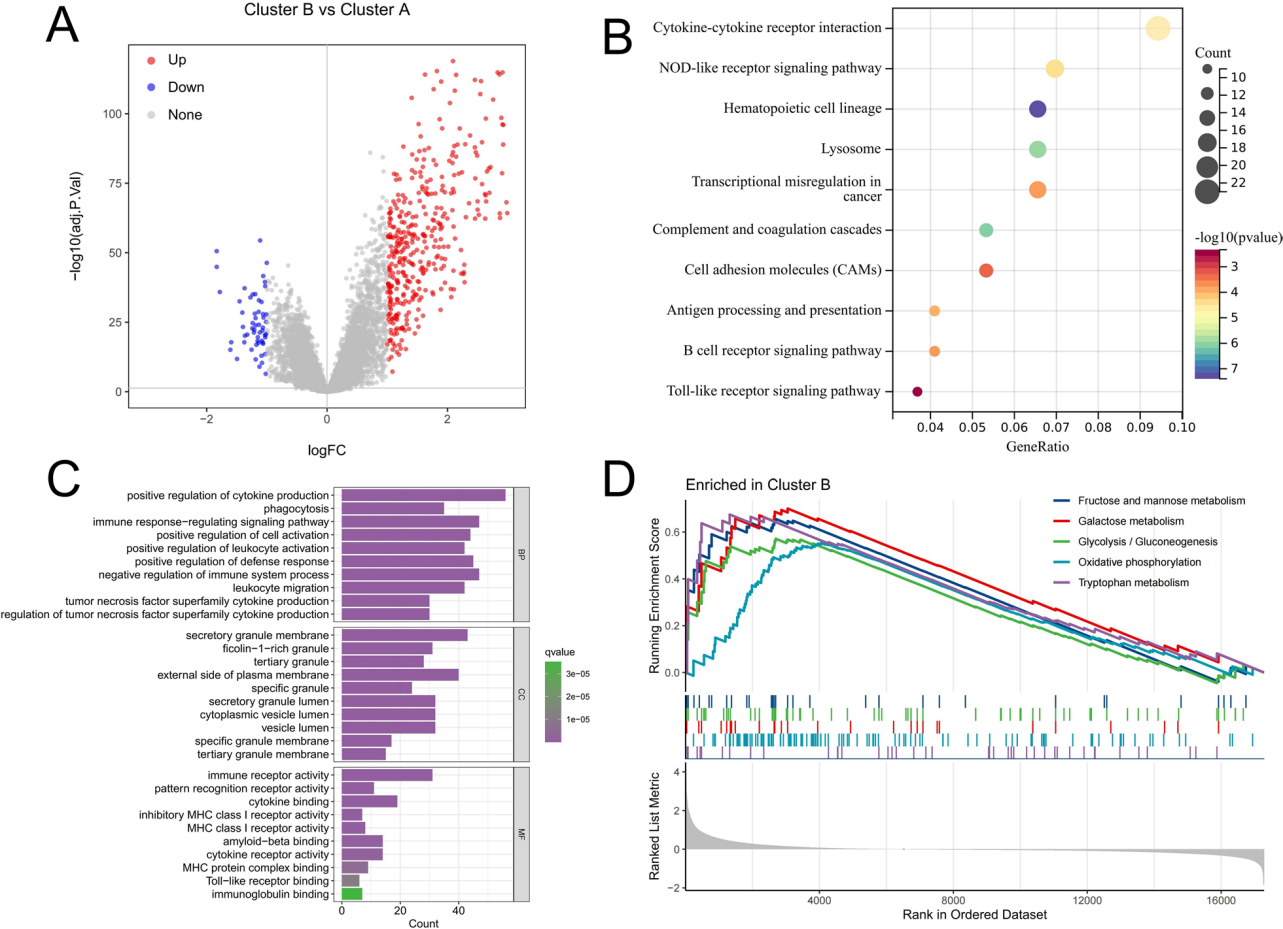
Functional analysis of DEGs between ICD molecular subtypes. **A** The volcano plot shows the differential expression characteristics of genes between molecular subtypes. Red and blue dots indicate genes with significantly upregulated expression in Cluster B and Cluster A, respectively. **B**, **C** KEGG (**B**) analysis and GO annotation (**C**) of DEGs between ICD molecular subtypes. **D** GSEA analysis revealed significantly enriched signaling pathways in Cluster B

To fully understand the potential biological functions of ICD-related subtypes in AML, we identified 444 DEGs associated with ICD-related subtypes through differential analysis. Volcano map shows that most DEGs are up-regulated in Cluster B (Fig. 4A). Then, we performed a functional enrichment analysis of DEGs. According to KEGG analysis, These ICD subtype-related DEGs are enriched in immune-related pathways such as cytokine-cytokine receptor interaction, NOD-like receptor signaling pathway, and cell adhesion molecules (CAMs) and antigen processing and presentation (Fig. 4B). The GO annotation showed that the main functions of these genes include positive regulation of cytokine production, immune response-regulating signaling pathway, and immune receptor activity (Fig. 4C). GSEA analysis showed that the activities of metabolism-related pathways such as fructose and mannose metabolism, galactose metabolism, glycolysis/gluconeogenesis, oxidative phosphorylation, and tryphan metabolism in cluster B were significantly higher (Fig. 4D). These results again suggest that ICD is significantly correlated with immune signaling, and that abnormal metabolic signaling may also contribute to poor prognosis in Cluster B patients by promoting malignant proliferation of tumor cells.

### Prognostic predictive value of ICD subtype-related DEGs

**Fig. 5:**
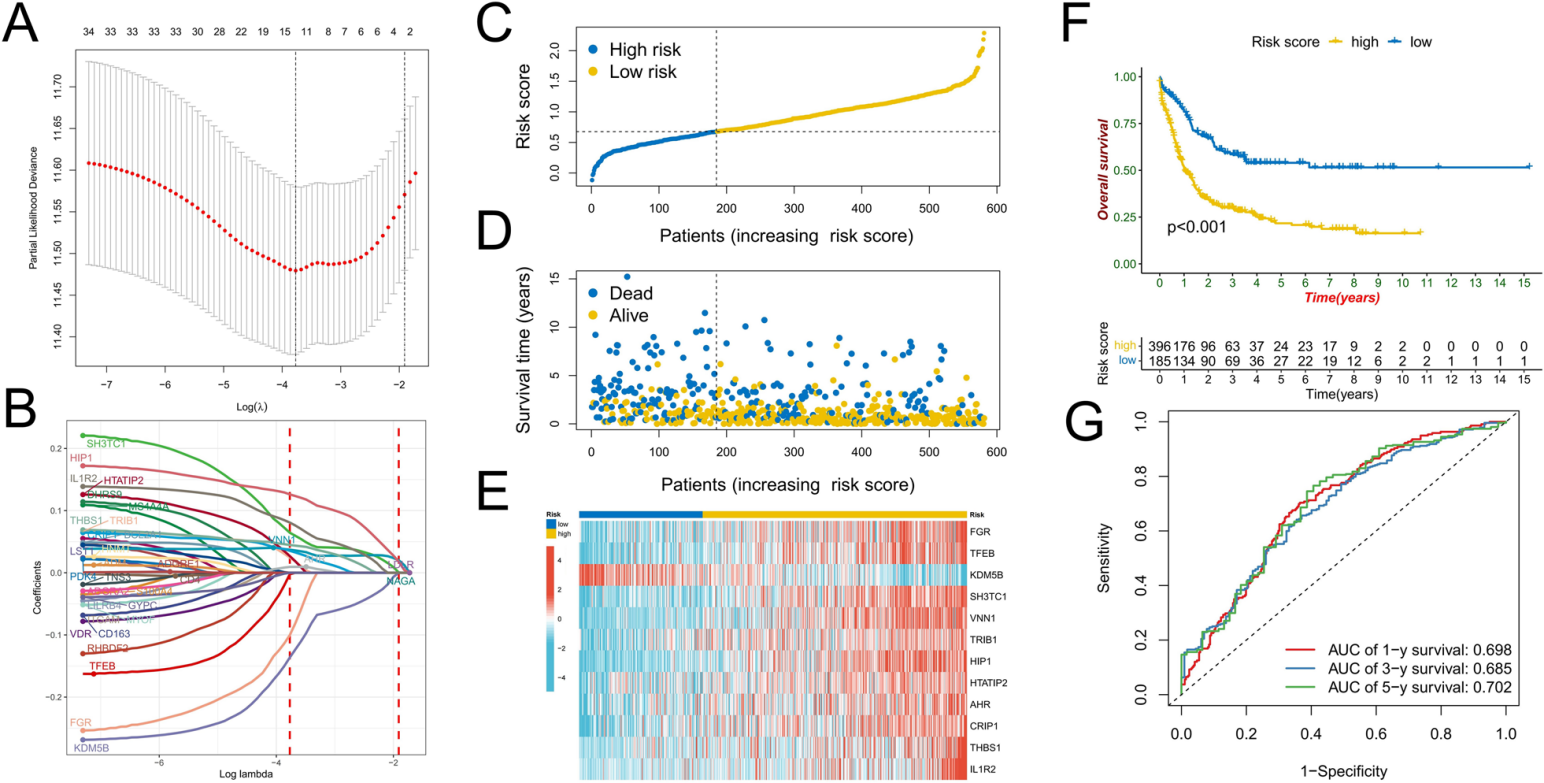
Construction of risk scoring model. **A** The penalty coefficient of the minimum tenfold cross-validation error point was calculated to determine the corresponding model gene. **B** Determination of model gene coefficients. **C**–**E** Based on the optimal cut-off value, patients in the meta-cohort were divided into high- and low-risk score groups (**C**); the survival status distribution (**D**), and model gene expression (b) in high- and low-risk score groups. **F** Survival analysis between high- and low-risk score groups. **G** time-dependent ROC curve analysis of risk scores in the meta cohort

**Fig. 6:**
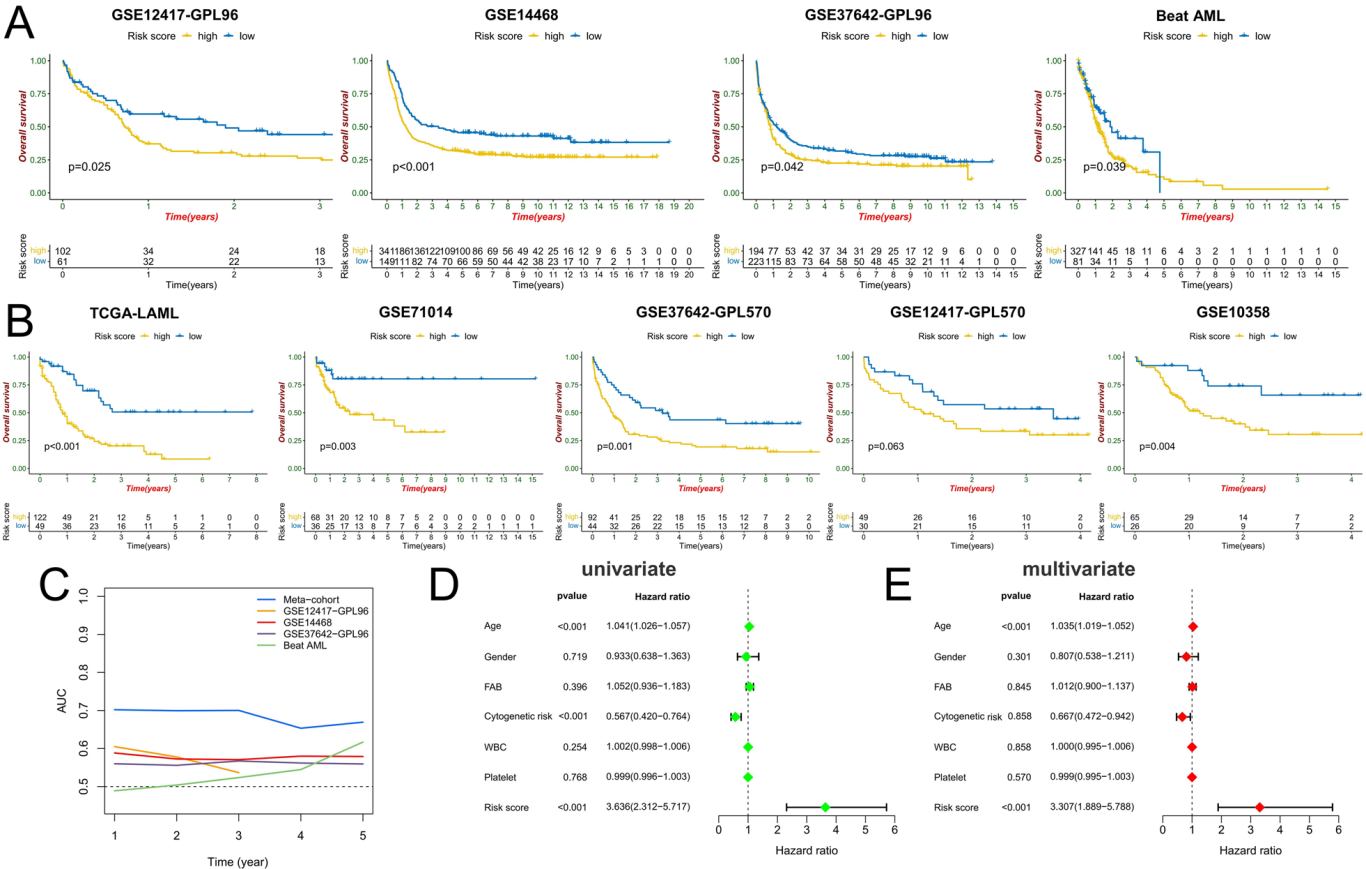
Validation of risk score model. **A**, **B** Survival analysis between high- and low-risk groups in the validation cohorts (**A**) and the constituent cohorts of the meta-cohort (**B**). **C** time-dependent ROC curve analysis of risk scores in the meta-cohort and the validation cohorts. **D**, **E** Univariate and multivariate Cox regression analysis of clinicopathological factors and risk score in the TCGA-LAML cohort

Through univariate Cox regression analysis, we found that 34 ICD subtype-related DEGs were significantly associated with AML prognosis (p < 0.05). LASSO regression analysis was used to further reduce dimensionality and construct a risk score model with FGR, TFEB, KDM5B, SH3TC1, VNN1, TRIB1, HIP1, HTATIP2, AHR, CRIP1, THBS1, and IL1R2 as model factors (Fig. 5A, B) (Additional file 1: Table S2). AML patients were divided into high-risk and low-risk groups based on the best cut-off value (Fig. 5C). Compared with the low-risk group, the high-risk group had significantly more patients who died (Fig. 5D). Except for KDM5B, the expression of model genes was significantly higher in the high-risk group (Fig. 5E). Survival analysis showed that the overall survival of the high-risk score group was significantly shorter than that of the low-risk score group (Fig. 5F). ROC curve analysis confirmed the prognostic prediction efficacy of the risk score model, and the AUC values for predicting 1-, 3-, and 5-year overall survival were 0.698, 0.685, and 0.702, respectively (Fig. 5G). In the nine AML cohorts including the meta-cohort, it was confirmed that the prognosis of patients in the high-risk group was significantly worse (p < 0.05) (Fig. 6A, B). The multi-cohort data confirmed the prognostic predictive value of the risk score model, which was confirmed by ROC curve analysis (Fig. 6C). The TCGA-LAML cohort contained more clinical data. Univariate and multivariate Cox regression analysis confirmed that the risk score was an independent factor in predicting the prognosis of AML (p < 0.001) (Fig. 6D, E).

### Potential molecular mechanisms affecting the prognosis of patients in different risk groups

**Fig. 7:**
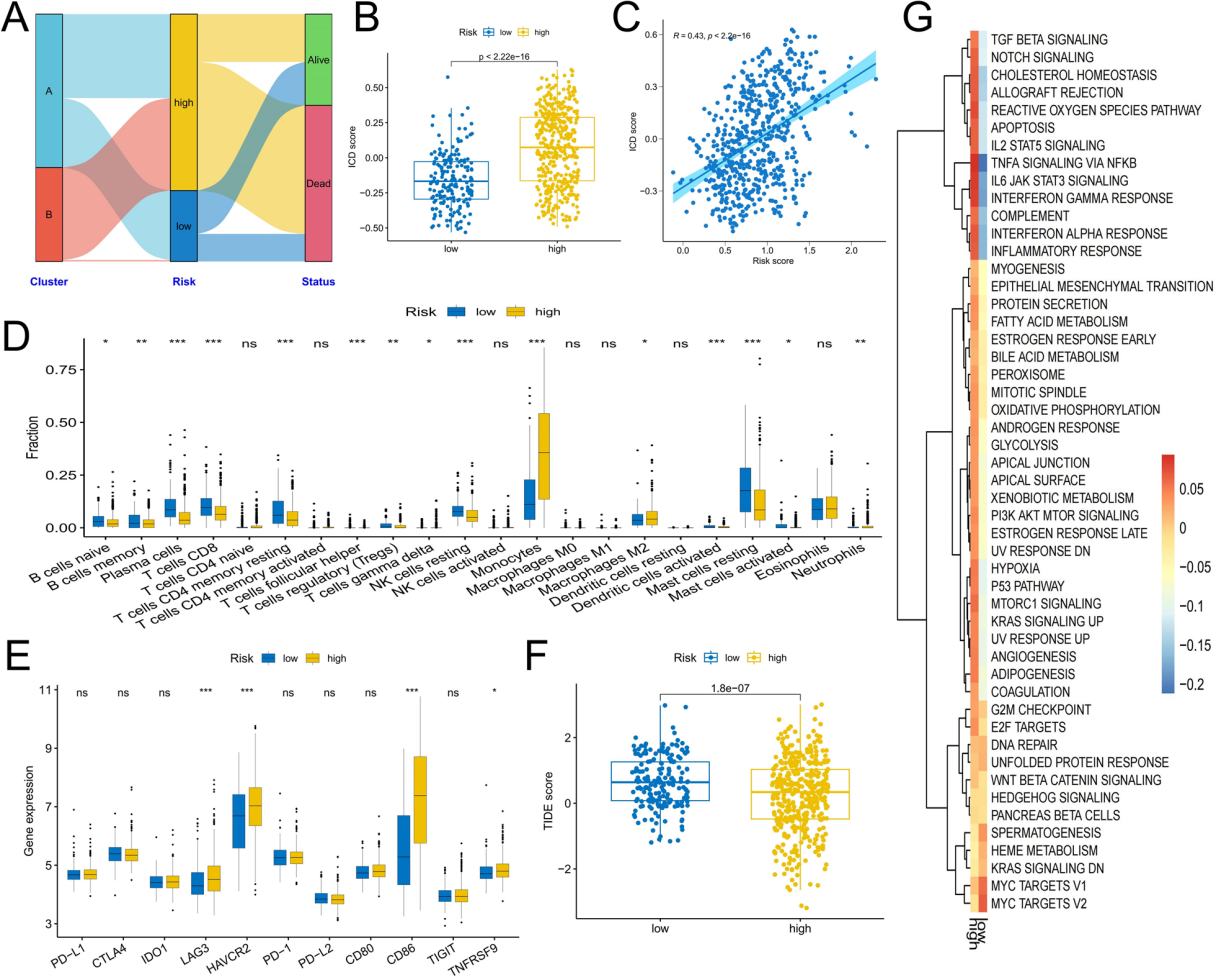
Differences in TME characteristics between high- and low-risk score groups. **A** Alluvial plots show the distribution of molecular subtypes, risk-score groups, and vital status of patients. **B**, **C** Differences in ICD scores between high- and low-risk groups and the correlation between risk scores and ICD scores. **D**–**G** Differences in immune cell infiltration (**D**), immune checkpoint expression (**E**), TIDE score (**F**), and tumor marker pathway enrichment score (**G**) between high- and low-risk groups

To better reveal the factors affecting the prognosis of patients in different risk groups, we systematically evaluated the differences in TME characteristics and clinicopathological factors between the two groups. The alluvial diagram shows that almost all patients in the low-risk group belong to cluster A, while patients in cluster B belong to the high-risk group, indicating that the risk score model further groups AML patients according to clinical outcomes based on ICD related molecular subtypes (Fig. 7A). The ICD score of patients in the high-risk group was significantly higher than that in the low-risk group, and the risk score was also significantly positively correlated with the ICD score, indicating that the risk score can also accurately reflect the ICD characteristics (Fig. 7B, C). The analysis of differences in immune cell infiltration and checkpoint expression showed that the difference between high-risk and low-risk groups was consistent with the difference between ICD-related subtypes, that is, the low-risk group showed increased infiltration of innate and adaptive immune cells, including Tregs, while the high-risk group showed up-regulated expression of immune checkpoints such as HAVCR2, LAG3, CD86 and TNFRSF (Fig. 7D, E). The low-risk group similarly showed a higher TIDE score (Fig. 7F). In addition, the activity of most tumor marker gene sets in Cluster A was higher than that in Cluster B (Fig. 7G). Combining these analysis results, we speculate that although the prognosis of low-risk patients is better, the infiltration of Tregs may hinder the immune effect of anti-tumor immune cells, and the high expression of immune checkpoints and active cancer-promoting signaling pathways may be the reasons for the poor prognosis of high-risk group.

### Differences in genomic traits and clinicopathological features between low- and high-risk groups

**Fig. 8:**
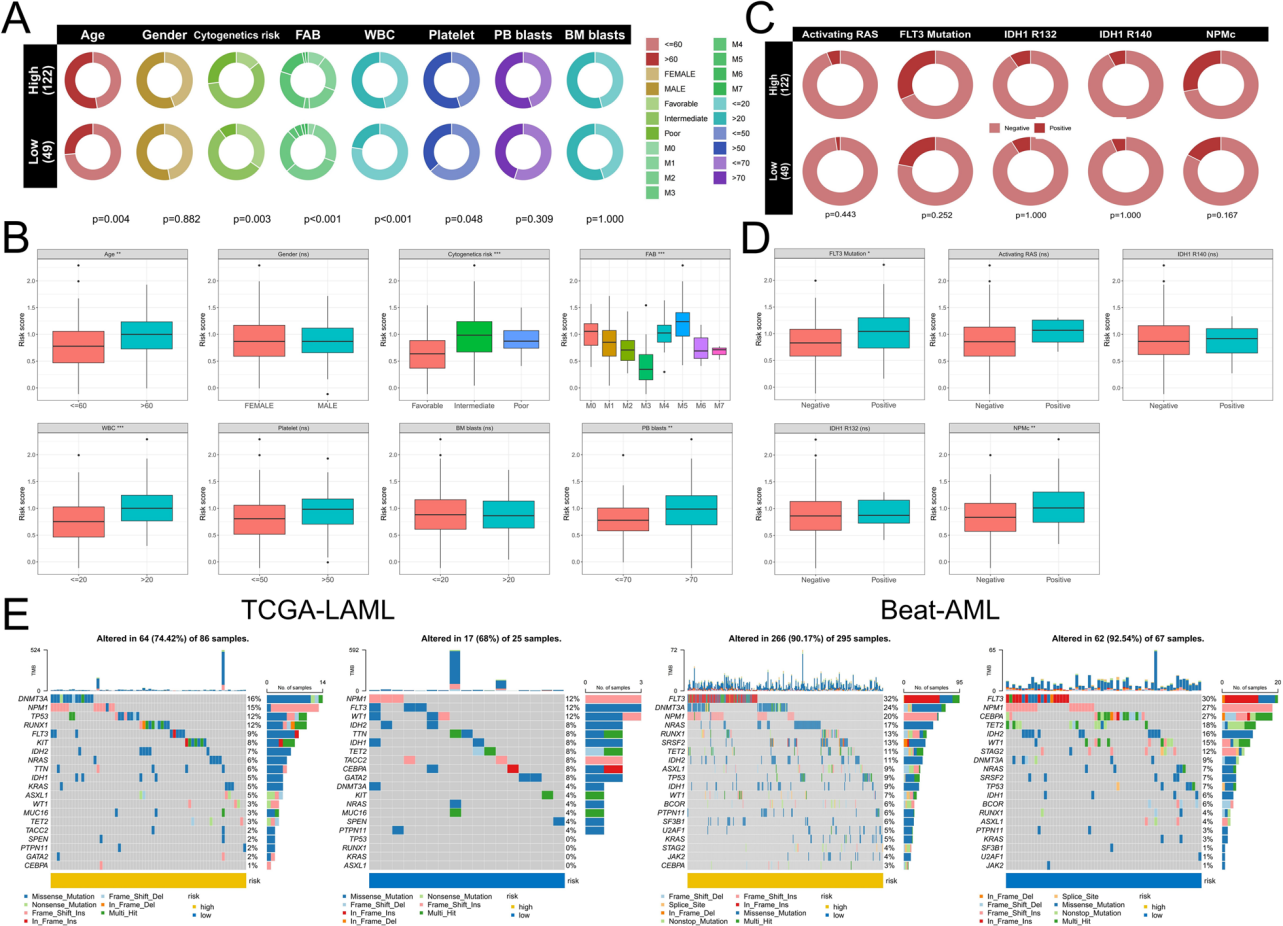
Differences in clinical characteristics and somatic mutations between high- and low-risk groups. **A**, **B** The differences in the proportion of conventional clinicopathological factors between high- and low-risk groups and the differences in risk scores between different clinicopathological factors were compared. **C**, **D** The difference in the proportion of somatic mutation positive and negative patients between high- and low-risk groups and the difference in risk scores between somatic mutation positive and negative patients. **E** Differences in overall somatic mutation frequency between high- and low-risk groups. FAB: French–American–British; WBC: white blood cell

We further analyzed the differences in clinicopathological characteristics between risk groups in the TCGA-LAML cohort. First, compared with the low-risk group, patients in the high-risk group were older (aged > 60 years), had poor cytogenetic risk, had more FAB classifications of M0, M4, and M5, and had a higher proportion of platelets and white blood cells (WBC) (Fig. 8A). Correspondingly, these patients also had higher risk scores (Fig. 8B). In addition, there was no significant difference in the proportion of patients with common somatic mutations between high-risk and low-risk groups (Fig. 8C), but the risk score of patients with negative FLT3 and NPM1 mutations was significantly lower than that of patients with positive (Fig. 8D). According to somatic mutation analysis, in the two cohorts (TCGA-LAML and Beat AML), FLT3, DNMT3A, NPM1, TP53, and RUNX1 mutations were most likely to occur in the high-risk group, and the mutated genes in the low-risk group mainly included FLT3, NPM1, CEBPA, TET2, IDH2, and WT1 (Fig. 8E). These results suggest that the differences in clinicopathological characteristics and somatic mutations may be important reasons for the differences in AML prognosis.

### Differences in chemosensitivity and response to immunotherapy between risk groups

**Fig. 9:**
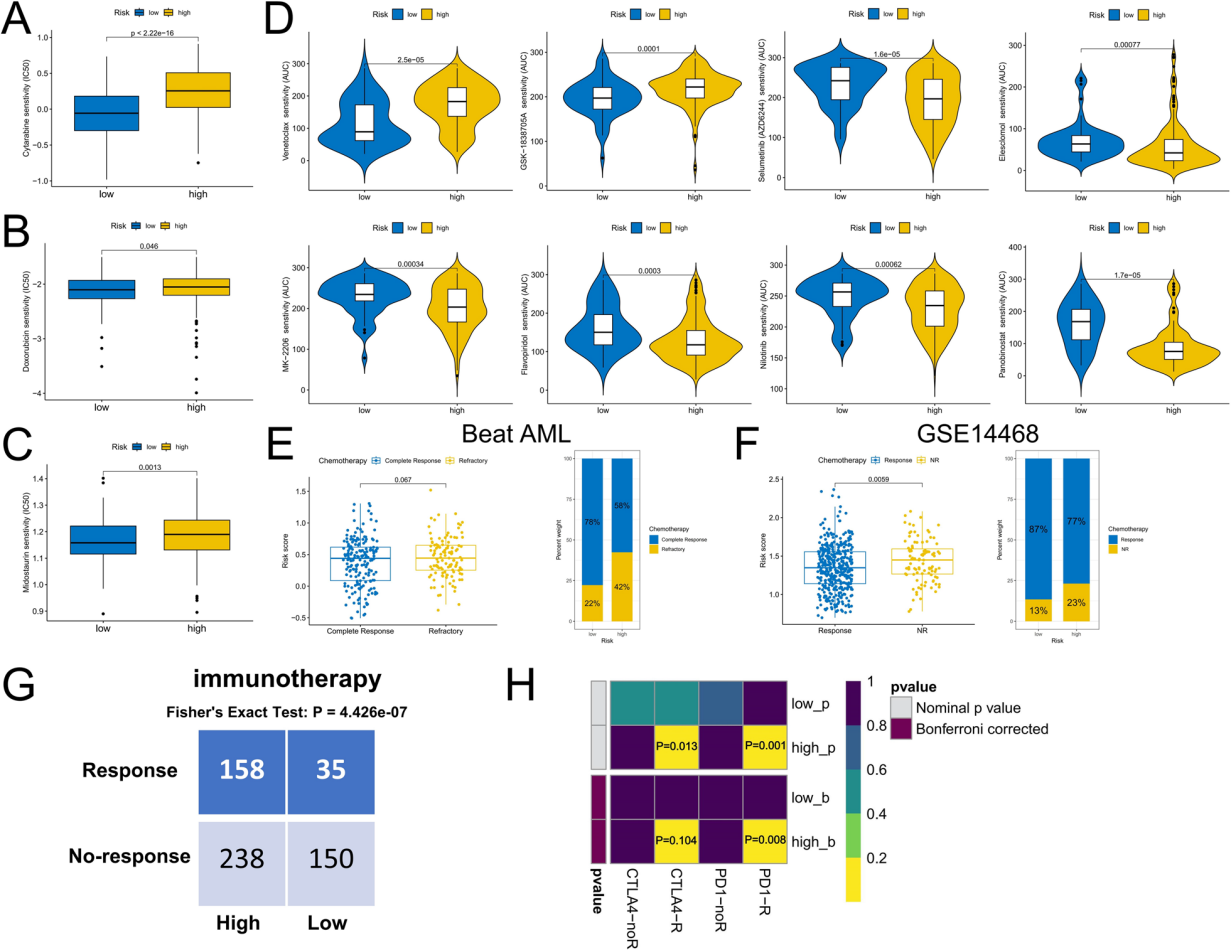
Differences in chemotherapy sensitivity and immunotherapy response between high- and low-risk groups. **A**–**C** Sensitivity prediction of cytarabine, doxorubicin, and midostaurin for AML in high- and low-risk groups. **D** Chemotherapeutic agents with differential sensitivity between risk groups in the Beat AML cohort. **E**, **F** The differences in risk scores of patients with or without response to induction chemotherapy and the proportion differences between risk groups. **G** Prediction of the proportion of patients with or without response to immunotherapy in different risk score groups. **H** Prediction of response to anti-PD-1 and anti-CTAL4 immunotherapy in different risk score groups

We predicted sensitivity to commonly used drugs in AML, with the lower-risk group having a lower IC50 to doxorubicin, cytarabine, and midostaurin and being more sensitive to them (Fig. 9A, C). Analysis of drug sensitivity data from ex vivo leukemia cells in the Beat AML cohort showed that the low-risk group was more sensitive to treatment with GSK-1838705A and Venetoclax. While the high-risk group was more sensitive to Elesclomol, Flavopiridol, MK-2206, Nilotinib, Panobinostat, and Selumetinib (AZD6244) (Fig. 9D). Moreover, in both Beat AML and GSE14468 cohorts, we observed that the proportion of patients who responded to induction chemotherapy was higher in the low-risk group than in the high-risk group, and that patients who did not respond to induction chemotherapy had significantly higher risk scores than those who did (Fig. 9E, F). We used the TIDE algorithm to predict the possibility of patients in different risk groups responding to immunotherapy, and the results showed that the high-risk group (40%, 158/396) was more likely to respond to immunotherapy than the low-risk group (19%, 35/185) (P = 4.426e−07) (Fig. 9G). We also used subclass mapping to compare the expression profiles of high- and low-risk groups with another dataset containing 47 melanoma patients who responded to immunotherapy [19]. Excitingly, in the meta-cohort, we observed that the high-risk group was more likely to respond to anti-PD-1 (P = 0.001) and anti-CTLA4 (P = 0.013) treatments (Fig. 9H). This may be related to the higher expression level of immune checkpoints in the high-risk group.

### The expression of risk score model genes was validated in a clinical real-world cohort

**Fig. 10:**
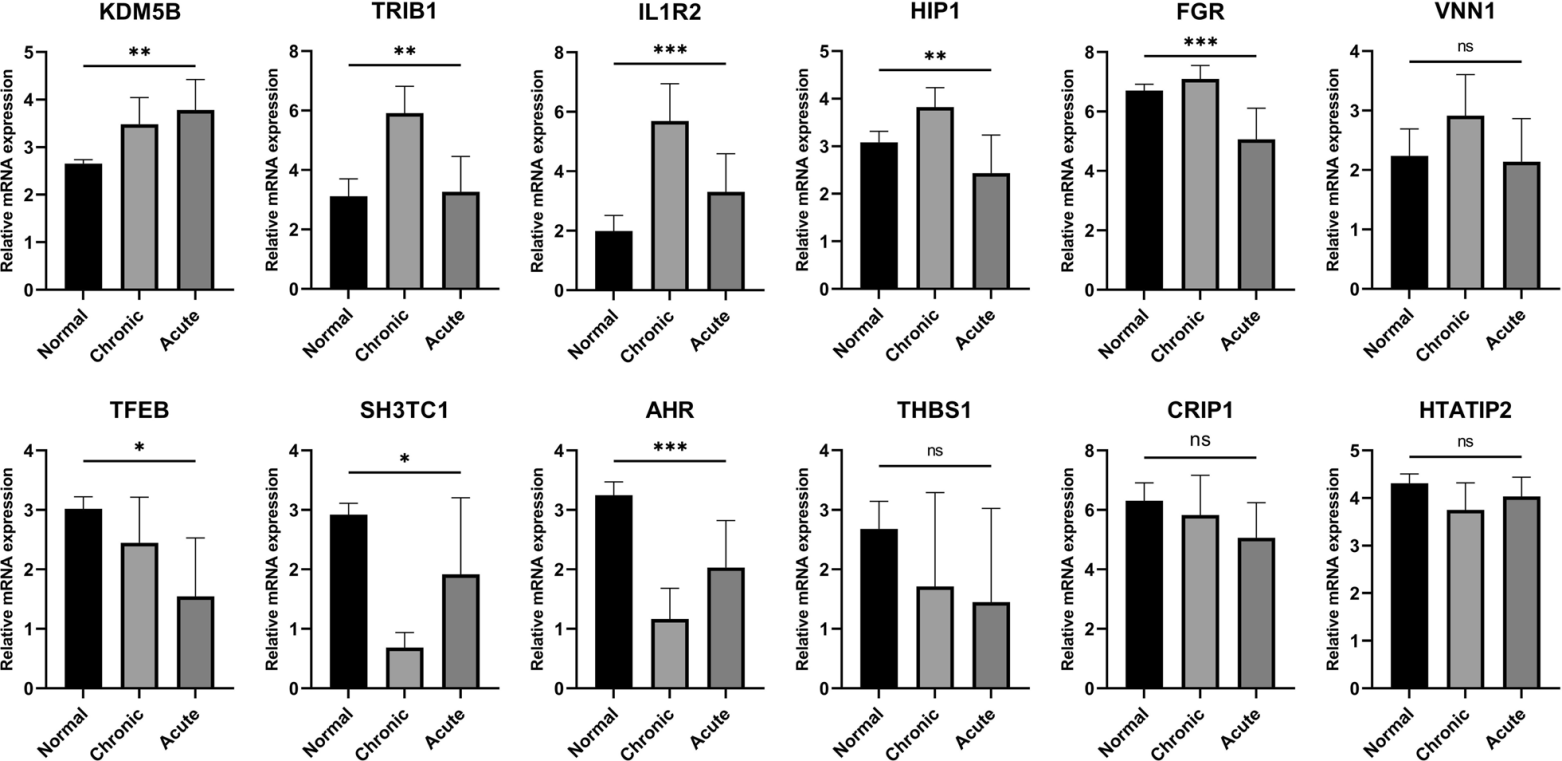
The clinical cohort was used to validate the expression of risk score model genes. Differences in the expression of risk-scoring model genes among normal samples, myeloid leukemia chronic-phase samples, and myeloid leukemia acute-phase samples

We collected five normal samples, five myeloid leukemia chronic-phase samples, and five myeloid leukemia acute-phase samples for transcriptome sequencing. Compared with the normal samples, the expression of KDM5B and IL1R2 was up-regulated in both chronic and acute phase samples, and the expression of TRIB1, HIP1, VNN1, and IL1R2 was only up-regulated in the chronic samples, The expression of FGR was up-regulated in chronic phase samples and down-regulated in acute phase samples, and the expression of TFEB, SH3TC1, and AHR was down-regulated in both chronic and acute phase samples. Moreover, there was no significant change in the expression of THBS1, CRIP1, and HTATIP1 (Fig. 10).

## Discussion

AML is a blood tumor with extensive molecular variation [1]. Its pathogenesis is complex, and the high heterogeneity of the disease often leads to poor prognosis [20]. At present, the treatment of AML still mainly relies on high-dose chemotherapy. Although the development of a variety of targeted therapeutics has benefited the treatment of AML [21], the frequent occurrence of relapse and adverse reactions has plagued clinical treatment due to the existence of various escape mechanisms [22], which also prompted people to explore new therapeutic methods, including immunotherapy [23].

Immunotherapy such as CAR-T cell therapy has made significant progress in hematological tumors, but due to the frequent occurrence of adverse reactions and the differences in individual characteristics, the impact on AML is not encouraging [24]. Therefore, the development of new therapeutic strategies to enhance the response of immunotherapy and chemotherapeutic drug sensitivity has shown important clinical value in improving the prognosis of AML. As a kind of regulatory cell death, ICD triggers an antigen-specific adaptive immune response by generating danger signals or DAMPs [6, 7]. For example, ICD can transform dying cancer cells into a "vaccine" to promote antitumor immunity by maturing dendritic cells, stimulating cytotoxic T lymphocytes, and enhancing NK cell cytotoxicity [5]. ICD plays an important anti-cancer role in a variety of cancers, such as prostate cancer [25], ovarian cancer [26] and colon cancer [27]. ICD contributes to the efficacy of chemotherapy and immunotherapy in solid cancer, and its impact on AML has only been partially explored. Previous studies have shown that the increased proportion of CD8+ T cells in the blood of AML patients receiving anthracycline consolidation therapy contributes to the efficacy of immunotherapy with histamine dihydrochloride (HDC) and IL-2, and improves the survival rate of patients, which is due to the increased responsiveness of immunotherapy due to ICD generated by consolidation therapy [28]. Chemotherapy or γ Irradiation also triggered the ICD of AML cells by translocating calreticulin to the plasma membrane, promoting the immunity and type I interferon-dependent survival of AML mice [29]. A variety of chemotherapy drugs such as etoposide and daunorubicin can also promote AML treatment by inducing ICD [30]. Recent studies have shown that ICD-related models have potential value in predicting the prognosis and treatment response of a variety of tumors [31, 32]. Leukemia-related studies have demonstrated that the activation of AMPK induces the development of ICD in AML [33]. γ-Mangostin effectively suppresses leukemia cells by inducing ICD, which is characterized by increased expression of HSP90B1, ANXA1, and IL1B [34]. Activation of CD47 triggers ICD in T-cell acute lymphoblastic leukemia [35]. However, the mechanism of ICD in AML, the interaction between ICD and TME, and the value of ICD as a biomarker in predicting clinical outcomes and treatment decisions of AML patients are not clear.

Our study revealed the heterogeneity of the expression landscape of ICDRGs in AML. Most ICDRGs are upregulated in AML and are associated with poor prognosis. There are significant differences in TME characteristics and prognosis between molecular subtypes identified based on ICDRG expression. The construction of the ICD scoring system quantified the molecular subtypes and revealed the potential interaction between ICD and TME. Cluster A has a lower ICD score and better prognosis, as well as a higher proportion of anti-tumor immune cells such as CD4+ T cells, CD8+ T cells, NK cells, and dendritic cells, but it is also accompanied by the enrichment of more Tregs. Several previous studies have shown that the frequency of Tregs in AML patients increases and is associated with poor prognosis [36]. Treg depletion can enhance the therapeutic effect of NK cells and CAR-T cells on AML [37, 38]. Therefore, eliminating Tregs may improve the efficacy of ICD induction therapy for Cluster A patients. Cluster B patients have worse clinical prognosis, high expression of immune checkpoints, and immune infiltration inhibition. The expression of ICDRGs, such as TLR4, was significantly upregulated in Cluster B, indicating that this molecular subtype may exhibit heightened sensitivity to DAMPs and therefore potentially benefit from immunotherapy aimed at inducing ICD. Induction of increased immune infiltration and inhibition of immune checkpoint expression may contribute to the ICD effect in Cluster B. In addition, the increased activity of metabolic pathways may also be one of the reasons that affect the clinical outcome of Cluster B patients.

Finally, our risk score model constructed based on ICD subtype-related gene expression can accurately predict the prognosis of AML patients, and patients in the high-risk group have worse clinical outcomes. The AUC values of 1-, 3-, and 5-year overall survival of AML patients predicted by the risk score model were 0.698, 0.685, and 0.702, respectively. Univariate and multivariate regression analysis also showed that risk score was an independent prognostic factor for AML. The prognostic predictive value of the risk score model was validated in nine AML cohorts. Analysis of clinical and biological differences between high and low-risk score groups revealed potential factors affecting the prognosis of AML patients. In the high-risk group, the immune cell infiltration was reduced, the high expression of immune checkpoints was up-regulated, and a higher proportion of older patients with adverse cytogenetic risk were included. The WBC and platelet count in the high-risk group were also increased. Additionally, we observed a high frequency of FLT3 and NPM1 mutations in both the high and low-risk groups, indicating their close association with the development and progression of AML. Our study also revealed that patients with FLT3 and NPM1 mutations had a higher risk score, suggesting a potential link to poor prognosis. However, due to the diversity and complexity of AML mutations, it may not be sufficient to predict patient prognosis based solely on a single gene mutation. Nevertheless, it is important to consider that targeted therapies directed at these specific mutations may benefit patients in the high-risk group. To better provide guidance on treatment strategies for patients in different risk groups, our analysis found that the low-risk group was more sensitive to common chemotherapy drugs such as cytarabine, doxorubicin, and midostaurin. The drug treatment data of ex vivo AML cells showed that GSK-1838705a and Venetoclax were more promising for the treatment of low-risk patients, while high-risk patients may benefit from the use of Elesclomol, Flavopiridol, MK-2206, nilotinib, Panobinostat, and Selumetinib. Because of the abnormal expression of immune checkpoints in the high-risk group, we predicted that patients in the high-risk score group would respond to anti-PD-1 and anti-CTLA4 treatment.

In conclusion, we revealed that there is a crosstalk between ICD and the occurrence and development of AML, and it is closely related to different immune cell infiltration and signaling pathway activity. The risk score model based on ICD subtype-related genes can be used as an independent factor to accurately predict the prognosis of AML patients, indicate the characteristics of TME, and provide guidance for the formulation of personalized treatment strategies for AML. Our study also has some limitations. Only through bioinformatics methods and clinical sample sequencing revealed the expression characteristics of ICDRGs, as well as the potential biological mechanism and prognostic value of ICD. We will take more in vivo and in vitro experiments to analyze the biological functions of ICDRGs in AML cells in future studies, and verify the prognostic value of the risk score model through a multicenter, larger sample real-world cohort.

## Conclusions

We systematically analyzed the expression characteristics of ICDRGs and compared the differences in clinical pathological features, immune infiltration, and molecular landscape between ICD related-classifications. The risk score model has considerable value in predicting AML prognosis and evaluating treatment response, and may help clinical doctors design personalized treatment strategies.

### Supplementary Information

**Additional file 1: Table S1.** Basic information of series used in this study. **Table S2.** LASSO regression model genes and coefficients.

## Data Availability

All data used in this work can be acquired from the Gene-Expression Omnibus (GEO; https://www.ncbi.nlm.nih.gov/geo/) and The Cancer Genome Atlas (TCGA) database (https://portal.gdc.cancer.gov/).

## References

[1] Shimony S, Stahl M, Stone RM (2023). Acute myeloid leukemia: 2023 update on diagnosis, risk-stratification, and management. Am J Hematol.

[2] Larsson CA, Cote G, Quintás-Cardama A (2013). The changing mutational landscape of acute myeloid leukemia and myelodysplastic syndrome. Mol Cancer Res.

[3] DiNardo CD, Perl AE (2019). Advances in patient care through increasingly individualized therapy. Nat Rev Clin Oncol.

[4] Casares N (2005). Caspase-dependent immunogenicity of doxorubicin-induced tumor cell death. J Exp Med.

[5] Krysko DV (2012). Immunogenic cell death and DAMPs in cancer therapy. Nat Rev Cancer.

[6] Galluzzi L (2020). Consensus guidelines for the definition, detection and interpretation of immunogenic cell death. J Immunother Cancer.

[7] Ahmed A, Tait SWG (2020). Targeting immunogenic cell death in cancer. Mol Oncol.

[8] Kawano M (2016). Dendritic cells combined with doxorubicin induces immunogenic cell death and exhibits antitumor effects for osteosarcoma. Oncol Lett.

[9] Fucikova J (2016). Calreticulin exposure by malignant blasts correlates with robust anticancer immunity and improved clinical outcome in AML patients. Blood.

[10] Lecciso M (2017). ATP release from chemotherapy-treated dying leukemia cells elicits an immune suppressive effect by increasing regulatory T cells and tolerogenic dendritic cells. Front Immunol.

[11] Garg AD, De Ruysscher D, Agostinis P (2016). Immunological metagene signatures derived from immunogenic cancer cell death associate with improved survival of patients with lung, breast or ovarian malignancies: a large-scale meta-analysis. Oncoimmunology.

[12] Wilkerson M, Hayes D (2010). ConsensusClusterPlus: a class discovery tool with confidence assessments and item tracking. Bioinformatics.

[13] Yu G, Wang LG, Han Y, He QY (2012). clusterProfiler: an R package for comparing biological themes among gene clusters. OMICS.

[14] Newman A (2015). Robust enumeration of cell subsets from tissue expression profiles. Nat Methods.

[15] Yoshihara K (2013). Inferring tumour purity and stromal and immune cell admixture from expression data. Nat Commun.

[16] Ritchie M (2015). limma powers differential expression analyses for RNA-sequencing and microarray studies. Nucleic Acids Res.

[17] Geeleher P, Cox N, Huang R (2014). pRRophetic: an R package for prediction of clinical chemotherapeutic response from tumor gene expression levels. PLoS ONE.

[18] Li SQ (2020). Transcriptome profiling reveals the high incidence of hnRNPA1 exon 8 inclusion in chronic myeloid leukemia. J Adv Res.

[19] Roh W (2017). Integrated molecular analysis of tumor biopsies on sequential CTLA-4 and PD-1 blockade reveals markers of response and resistance. Sci Transl Med.

[20] Nair R, Salinas-Illarena A, Baldauf H (2021). New strategies to treat AML: novel insights into AML survival pathways and combination therapies. Leukemia.

[21] Döhner H, Wei A, Löwenberg B (2021). Towards precision medicine for AML. Nat Rev Clin Oncol.

[22] Hackl H, Astanina K, Wieser R (2017). Molecular and genetic alterations associated with therapy resistance and relapse of acute myeloid leukemia. J Hematol Oncol.

[23] Grosso D, Hess R, Weiss M (2015). Immunotherapy in acute myeloid leukemia. Cancer.

[24] Haslauer T, Greil R, Zaborsky N, Geisberger R (2021). CAR T-cell therapy in hematological malignancies. Int J Mol Sci.

[25] Wang X (2020). Targeting STAT3 enhances NDV-induced immunogenic cell death in prostate cancer cells. J Cell Mol Med.

[26] Lau TS (2020). Paclitaxel induces immunogenic cell death in ovarian cancer via TLR4/IKK2/SNARE-dependent exocytosis. Cancer Immunol Res.

[27] Ruan H, Leibowitz BJ, Zhang L, Yu J (2020). Immunogenic cell death in colon cancer prevention and therapy. Mol Carcinog.

[28] Aurelius J (2019). Anthracycline-based consolidation may determine outcome of post-consolidation immunotherapy in AML. Leuk Lymphoma.

[29] Chen X, Fosco D, Kline DE, Kline J (2017). Calreticulin promotes immunity and type I interferon-dependent survival in mice with acute myeloid leukemia. Oncoimmunology.

[30] Ocadlikova D, Iannarone C, Redavid AR, Cavo M, Curti A (2020). A screening of antineoplastic drugs for acute myeloid leukemia reveals contrasting immunogenic effects of etoposide and fludarabine. Int J Mol Sci.

[31] Wang X (2021). An immunogenic cell death-related classification predicts prognosis and response to immunotherapy in head and neck squamous cell carcinoma. Front Immunol.

[32] Decraene B (2022). Immunogenic cell death and its therapeutic or prognostic potential in high-grade glioma. Genes Immun.

[33] Mondesir J (2023). AMPK activation induces immunogenic cell death in AML. Blood Adv.

[34] Long ZJ (2023). Dietary γ-mangostin triggers immunogenic cell death and activates cGAS signaling in acute myeloid leukemia. Pharmacol Res.

[35] Uscanga-Palomeque AC (2019). CD47 agonist peptide PKHB1 induces immunogenic cell death in T-cell acute lymphoblastic leukemia cells. Cancer Sci.

[36] Szczepanski MJ (2009). Increased frequency and suppression by regulatory T cells in patients with acute myelogenous leukemia. Clin Cancer Res.

[37] Zhou Q (2009). Depletion of endogenous tumor-associated regulatory T cells improves the efficacy of adoptive cytotoxic T-cell immunotherapy in murine acute myeloid leukemia. Blood.

[38] Rooney CM (2014). Can Treg elimination enhance NK cell therapy for AML?. Blood.

